# Cavitand Complexes in Aqueous Solution: Collaborative Experimental and Computational Studies of the Wetting, Assembly, and Function of Nanoscopic Bowls in Water

**DOI:** 10.1021/acs.jpcb.0c11017

**Published:** 2021-03-02

**Authors:** Henry S. Ashbaugh, Bruce C. Gibb, Paolo Suating

**Affiliations:** † Department of Chemical and Biomolecular Engineering, Tulane University, New Orleans, Louisiana 70118, United States; ‡ Department of Chemistry, Tulane University, New Orleans, Louisiana 70118, United States

## Abstract

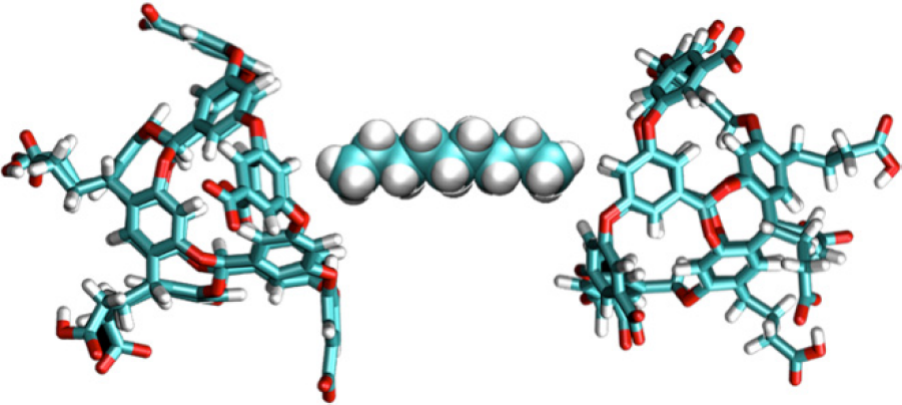

Water is the dominant liquid on Earth. Despite this, the main focus of supramolecular chemistry research has been on binding and assembly events in organic solvents. This arose because it is more straightforward to synthesize organic-media-soluble hosts and because of the relative simplicity of organic solvents compared to water. Nature, however, relies on water as a solvent, and spurred by this fact, supramolecular chemists have recently been making forays into the aqueous domain to understand water-mediated non-covalent interactions. These studies can benefit from the substantial understanding of the hydrophobic effect and electrostatic interactions developed by physical chemists. Nearly 20 years ago, the Gibb group first synthesized a class of water-soluble host molecules, the deep-cavity cavitands, that possess non-polar pockets that readily bind non-polar moieties in aqueous solution and are capable of assembling into a wide range of complexes with distinct stoichiometries. As such, these amphipathic host species are ideal platforms for studying the role of negatively curved features on guest complexation and the structural requirements for guided assembly processes driven by the hydrophobic effect. Here we review the collaborative experimental and computational investigations between Gibb and Ashbaugh over the past 10 years exploring questions including the following: How does water wet/solvate the non-polar surfaces of non-polar pockets? How does this wetting control the binding of non-polar guests? How does wetting affect the binding of anionic species? How does the nature and size of a guest size impact the assembly of cavitand hosts into multimeric capsular complexes? What are the conformational motifs of guests packed within the confines of capsular complexes? How might the electrostatic environment engendered by hosts impact the properties and reactivity of internalized guests?

In 2001, Gibb et al. demonstrated that a benzal-bridged resorcin[4]arene could be rigidified by an 8-fold Ullman biaryl ether coupling.^1^ The resulting deep-cavity cavitand (**1**, Figure 1) possessed (1) an enforced, non-polar pocket ∼8 Å deep and ∼8 Å at the portal and (2) a wide rim primarily comprised of aromatic rings. Because the composition of the host and the organic media it can be dissolved in are similar, guest complexation was relatively weak. Complexation was largely driven by two factors: (1) complementarity between the shapes of the host and the guest and (2) the four, inward pointing benzal hydrogens of **1** which could form hydrogen bonds to bound guests. Thus, ideally sized guests such as adamantane derivatives were the best guests, and within this class, halogenated derivates were the strongest binders because the halogen atom of the bound guest can form four C–H···X–R hydrogen bonds with the host.

**Figure 1:**
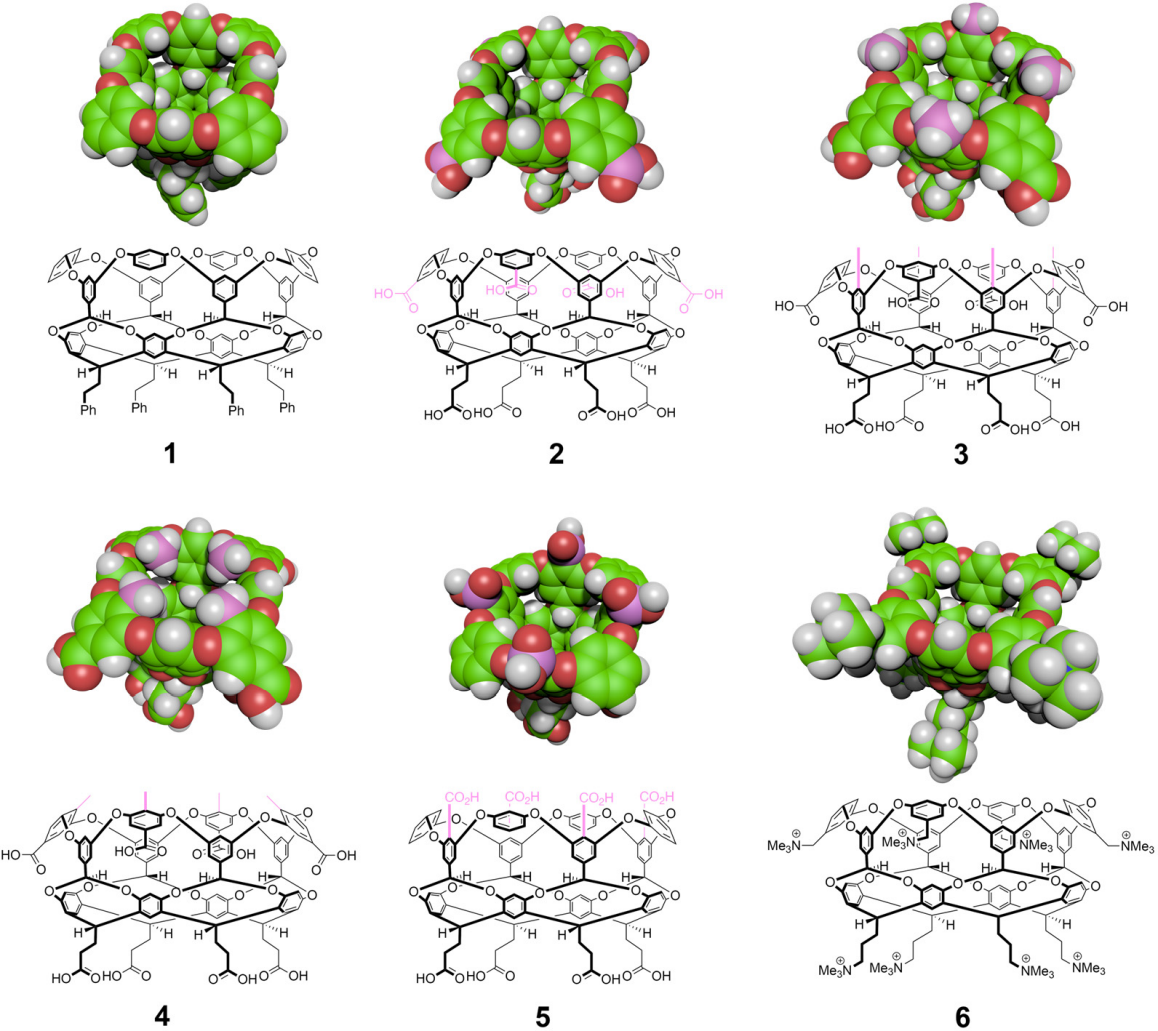
van der Waals and chemical structural representations of cavitand hosts **1**–**6** discussed here. Each of the hosts in this figure have been synthesized. For orientation purposes, the carboxylic acids and methyl groups of **2**–**5** are highlighted in pink in both the Chemdraw and space-filling representations. The hosts are as follows: **1** is the first deep-cavity cavitand synthesized by Gibb; **2** is deep-cavity cavitand octa-acid (OA); **3** is tetra-*exo*-methyl octa acid (TExMOA); **4** is tetra-*endo*-methyl octa acid (TEMOA); **5** is *exo*-octa acid; and **6** is referred to as the positand, which corresponds to host **2** with its carboxylic acid units replaced by quaternary ammonium groups. van der Waals structures were generated using ePMV for Cinema4D.^86^

A slightly adapted synthesis led to the so-called octa-acid (**2**, Figure 1), the first fully water-soluble deep-cavity cavitand.^2^ With a hydrophilic exterior coating of eight carboxylic acids, dissolution in basic media yields the corresponding octa-carboxylate host. Other water-soluble hosts have been since formed (Figure 1), but host **2** was the archetype that allowed many of the unusual properties of these hosts to be identified. Specifically, the hydrophobic effect not only drives the formation of 1:1 host–guest complexes (host–guest complexes noted as *X*:*Y*, where *X* is the number of hosts and *Y* is the number of guests in a complex) but also can drive the assembly of the hosts into dimeric, tetrameric, or hexameric assemblies containing one to three guest molecules within their yocto-liter inner spaces.^3−6^ As we describe in more detail below, these assembled capsules can be utilized for bringing about the separation of molecules from mixtures and as yocto-liter reaction vessels for the precision control of chemical conversions.^7−10^ Intimately tied to these properties is the solvation of the host, and in particular the solvation of its non-polar pocket and non-polar rim that respectively contribute to the binding of guests and the assembly of the host into capsules. Solvation of the pocket is of considerable interest because of the many open questions surrounding the hydration of tight “nooks and crannies” on the surface of proteins and other biomacromolecules, and correspondingly how hydration can drive function.

In 1973, Stillinger predicted that water will pull away from and dewet the surface of a nonattractive, hard sphere solute in aqueous solution as it grows in size, leaving only the whisper of a vapor-like layer in contact with the solute.^11^ This prediction is the direct result of the statistical mechanically exact “wall theorem” that dictates the density of the solvent in contact with a hard wall is determined by the ratio of the bulk pressure divided by the ideal gas law prediction or the solvent pressure using the actual solvent density, amounting to contact densities over 1000 times smaller than the bulk solvent for liquids at coexistence. Huang and Chandler subsequently predicted that turning on ubiquitous van der Waals attractions can collapse the vapor layer in contact with the wall.^12^ This predicted dewetting phenomenon is thought to play a major role in driving the interactions between hydrophobic surfaces in water, giving rise to interfacial forces between dewetted surfaces that drive them together.^13−16^ Simulations performed by Berne and co-workers have demonstrated dewetting as playing a role in the final steps of protein folding^17^ and in the formation of quarternary structure in protein assemblies,^18^ while simulations by Hummer and co-workers have found that dewetting of protein surfaces and internalized cavities can play a role in their function.^19−21^ Ashbaugh demonstrated the dewetting of hard spheres anticipated by Stillinger and showed its connections with the bulk interfacial tension of the liquid/vapor interface.^22−26^ Moreover, Ashbaugh has shown from simulations that attractive interactions do indeed suppress surface dewetting.^22,27^ Sarupria and Garde have subsequently found that, while van der Waals attractions enable rewetting of non-polar surfaces, solvent density fluctuations at those surfaces closely resemble those in the vapor highlighting water’s apprehension with hydrating extended hydrophobic domains.^28^

A majority of simulation studies of hydrophobic hydration have focused on convex, positively curved surfaces akin to the shape of small solutes, rather than on concave, negatively curved surfaces such as those found in protein binding sites and the hosts discussed here. An important exception to this are the simulations by Setny and co-workers of an idealized, hemispherical non-polar pocket in water. They found that water freely fluctuates between all possible hydration states from zero to 10 waters in the pocket,^29−31^ in agreement with Sarupria and Garde’s results regarding density fluctuations.^28^ In Setny’s simulations, the approach of a ligand to the pocket induced the free energy landscape of water to become bimodal, vacillating between a wet “liquid-like” and dry “vapor-like” state. Upon ligand binding, water was displaced from the pocket and the dewetted state became dominant. This dewetting-mediated binding, or triggered dissociative mechanism of guest exchange, was found to be driven by a favorable association enthalpy resulting from waters gaining attractive interaction upon release from the pocket. The entropy of association, on the other hand, opposed ligand binding. This dewetting driven interaction in water is distinct from that which typically comes to mind when discussing processes driven by the hydrophobic effect, i.e., that central to such phenomena is an entropic release of unfavorably structured water in the hydration shell of non-polar moieties in solution. Such processes are often termed as being driven by the classic hydrophobic effect. In contrast, host–guest binding processes in water are typically enthalpically driven associations and are frequently described as being driven by the nonclassical hydrophobic effect. This phenomenon has been experimentally observed for guests binding to proteins and host–guest species in water, supporting these simulation observations.^32−34^ Nau and co-workers have found a strong correlation between the number of hydrogen bonds water forfeits when it enters a non-polar confinement, as determined from simulation and experimental guest binding constants,^35,36^ although this hypothesis does not account for the bimodal equilibrium between wet and dry states observed by Setny. Nevertheless, taken together, these observations suggest complexations with non-polar pockets are distinct from the classic picture of hydrophobic association.^37^

Finding mutual research interests in hydrophobicity-driven complexation and assembly phenomena in aqueous solution, Ashbaugh and Gibb established a computational and experimental collaboration on the formation and properties of supramolecular complexes of deep-cavity cavitands in water. The range of cavitand hosts available (e.g., Figure 1) and their assembly into capsular complexes make them ideal for probing the role of curvature on driving supramolecular events in water. Following preliminary work on simulating competitive adsorption of idealized non-polar solutes,^38^ this collaboration has expanded to examine a wide range of issues, including the following: wetting/dewetting of cavitand pockets in water; assembly of cavitands and alkanes into a wide range of complexes that depend on the functionalization of the host portals and length of the encapsulated guests; packing of guests within host complexes; anion binding to host pockets at the root of salting-in Hoffmeister effects; and the encapsulated guest reaction catalysis driven by the electrostatic environment engendered by the host. Below, we detail the major experimental observations made regarding cavitand host–guest interactions in water by the Gibb group and the molecular insights gained from complementary computational work performed by the Ashbaugh group. We conclude with an outlook toward possible future directions for this mutually enriching collaboration.

## Wetting of Cavitand Pockets in Aqueous Solution

**Figure 2:**
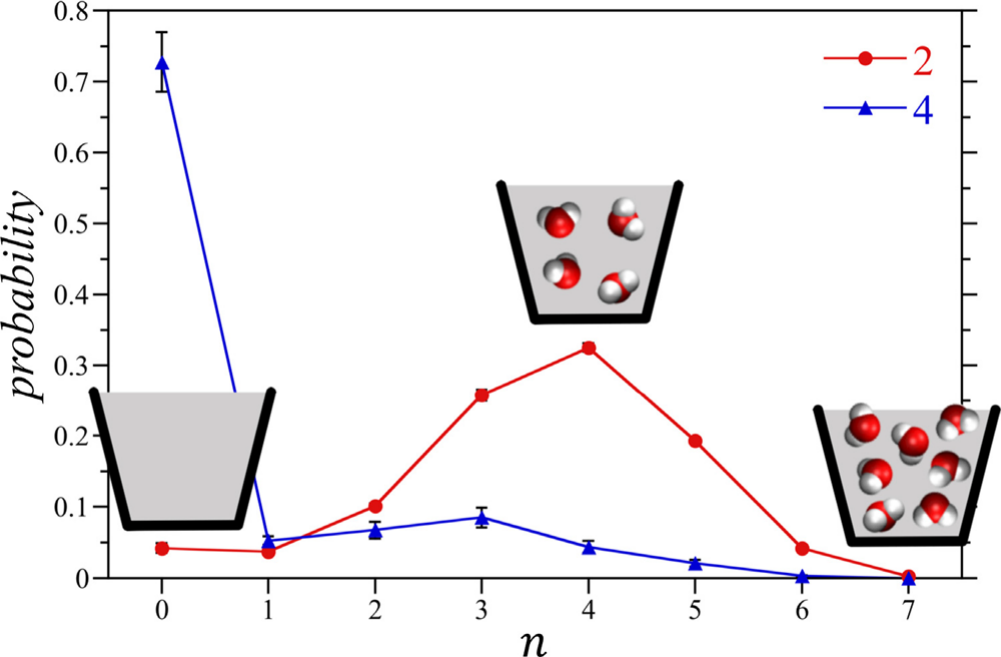
Probability of observing *n* waters within the non-polar pocket of hosts **2** and **4** at 25 °C and atmospheric pressure as determined from molecular simulation. The wet, liquid-like and dry, vapor-like pocket hydration states lie to the right and left of the probability minimum at *n* = 1 water in the pocket. Simulation error bars indicate one standard deviation, though most are comparable to the figure symbols. Adapted with permissions from ref (40). Copyright 2020 Springer Nature.

The first molecular simulations of deep-cavity cavitands in water focused on hydration of their binding pocket in the absence of added guests. Simulations performed by Ewell, Gibb, and Rick^39^ of host **2** in aqueous solution found that approximately four waters on average reside within the cavitand completely filling the non-polar pocket. The probability distribution of observing *n* waters in the pocket fluctuates from 0 to 8 waters, with transitions between occupation states occurring on ps time scales (Figure 2). The waters inside the pocket, however, were found to be more energetically frustrated relative to those in the bulk. The energetic absorption penalty is largely attributable to water losing approximately one hydrogen bond upon entering the pocket (dropping from 3.6 to 2.6 hydrogen bonds on average outside versus inside the pocket). These losses are compensated in part by gains in attractive van der Waals interactions of water with the inner walls of the cavitand. Moreover, the decrease in hydrogen bonding frees the absorbed waters to enjoy more configurational freedom, resulting in a concurrent increase in the entropy of the bound waters that favors absorption. While water spontaneously fills the pocket of host **2**, placing an ethane molecule—a model hydrophobic guest—just above the mouth of the host pocket induces a dewetting transition, with all of the waters spontaneously exiting the pocket even before the guest enters. It may then be concluded that this triggered dissociative mechanism arises because bound waters sit near the edge of thermodynamic stability, such that minor perturbations in their ability to hydrogen bond with bulk waters triggers dewetting to the dry state before the ethane guest binds.

The prediction that guests in the proximity of the portal of a cavitand can destabilize waters in the pocket begs the question: how might this be demonstrated experimentally? We have subsequently shown that the stability of water within this family of cavitands is sensitive to the functional groups around the binding site’s portal.^40^ In the case of tetra-*exo*-methyl octa acid (**3**, TExMOA, Figure 1) in which the portal is ringed by four methyl groups that marginally deepen the pocket but otherwise do not affect the aperture of the portal, water was shown from molecular simulations to wet the host pocket in a manner analogous to **2** with an average pocket occupancy of ∼4 waters. This situation is very different in the case of tetra-*endo*-methyl octa acid (**4**, TEMOA, Figure 1), however. Here, the four methyl groups ringing the portal somewhat narrow the pocket’s entrance, and our simulations predicted that water spontaneously evacuates the cavitand so that >70% of the time the pocket is dry (Figure 2). This drying is reminiscent of the dewetting transition observed for host **2** as ethane approaches the pocket^39^ and undoubtedly arises from the same thermodynamic drive.

The predicted drying of host **4**’s pocket was subsequently confirmed using densimetry experiments. Specifically, the partial molar volumes of hosts **2**–**4** were determined by a thermodynamic analysis of the aqueous-solution densities of these cavitands over a range of concentrations down to infinite dilution. The partial molar volume difference between hosts **3** and **2** was 73 ± 7 cm^3^ mol^–1^, corresponding to 4 times the volume increment of an individual methyl unit in water. The volume difference between hosts **4** and **2**, on the other hand, was 162 ± 12 cm^3^ mol^–1^, significantly greater than the volume of the methyl groups. The partial molar volume difference between hosts **4** and **3** of 89 ± 13 cm^3^ mol^–1^, subsequently, corresponds to the extra volume of host **4**. Considering that an individual liquid water molecule has a molar volume of 18 cm^3^ mol^–1^, this corresponds to the volume of four to five water molecules ejected from the host pocket. These experimental volume differences strongly correlate with those determined from simulation, although these results suggest that the pocket of host **4** is even drier than predicted. Further analysis of the simulation results demonstrated that the volumes of hosts **3** and **4** are the same when compared on the basis of the number of waters residing within their pockets. Since the partial molar volumes of all of the cavitands were shown to decrease with increasing pocket occupation, it follows that the anomalously large volume of host **4** is a direct result of the distribution of pocket occupancy states of host **4** tilted toward drier (i.e., lower occupancy) states compared to either host **2** or host **3**. Analogously, *exo*-octa acid (**5**, Figure 1) possessing four hydrophilic carboxylate groups around the rim was also found to have the same probability of approximately four waters in the pocket as **2**.^41^ In the case of **5**, however, the *exo* carboxylates stabilize a network of water molecules at the rim via hydrogen bonding, leading to a dewetting free energy that is 2.2 kJ mol^–1^ greater than **2**.

A natural question following from these observations is, what is the impact of host pocket drying on cavitand–guest association? Concomitant isothermal titration calorimetry (ITC) experiments examined the thermodynamics of binding of a series of sodium alkanoates, from *n*-hexyl- to *n*-decyl-, with hosts **2** and **4**.^40^ These experiments demonstrated that guest binding was generally stronger to host **4** than host **2**. Moreover, host–guest association was enthalpically favored and entropically disfavored—a signature of the nonclassical hydrophobic effect. Given that evacuation of the host pocket is a necessary step for guest binding, it may be hypothesized that the thermodynamics of cavitand drying plays a significant role in association. Examination of the free energies of emptying the pockets of hosts **2** and **4** using molecular simulations, unsurprisingly, found that the free energy of drying host **4** was lower than that of **2**.^40^ More importantly, pocket drying was enthalpically favorable and entropically unfavorable. The thermodynamic signatures of the drying of host **4** compared to **2** are similar in magnitude and of the same sign as that associated with sodium alkanoate binding, providing strong empirical evidence that pocket drying is a significant contributor to the association process, although questions do persist. Notably, it is unclear at the molecular level why the experimentally determined heat capacity of sodium alkanoate binding to host **4** is more negative than that to host **2**. It should also be noted in passing that other types of guest molecules display similar thermodynamic signatures upon binding to OA **2** and TEMOA **4**. For example, in studies examining the salting-in Hofmeister effect, it was observed that a range of polarizable anions (I^–^, ClO_4_^–^, PF_6_^–^, etc.) bind exothermically to the hydrophobic pockets of **2** and **4**, despite the two hosts being at least nominally, if not literally, octa-anionic.^42,43^

The drying of cavitand pockets can be attributed to destabilization of the liquid state of water induced by confining non-polar surfaces. It has been shown that the interfacial free energy of wetting a macroscopic hydrophobic surface can induce large-scale density fluctuations in water that tilt the equilibrium toward the vapor phase.^44,45^ When sandwiched between two flat, non-polar surfaces, water will spontaneously evacuate the slit as a result of capillary forces once the separation between surfaces falls below a critical distance.^13−15^ The driving force for drying can be exacerbated for negatively curved pits and depressions, as in the case of hosts such as cavitands, cyclodextrins, and cucurbiturils.^19,29,46,47^ Examination of the water occupation probabilities of the pockets of **2** and **4** from simulation finds that the distribution is bimodal (Figure 2), with peaks at zero and approximately four occupying waters separated by a probability minimum between these two states at a pocket occupancy of one water molecule. In the case of hosts **2** and **3**, the *n* = 4 peak is dominant, while, for host **4**, the *n* = 0 peak is dominant. The tipping of equilibrium from wet to dry states from hosts **2** and **3** to host **4** results from the *endo*-methyl groups reducing the aperture of the portal and reducing the free energy penalty for stabilizing a liquid–vapor interface across the entrance. While referring to zero waters in a cavitand as a vapor state and four waters in a cavitand as a liquid state should rightfully raise concern due to the inherent scale differences between the pocket of a cavitand and a bulk phase, we have previously shown that the macroscopic theory of capillary evaporation can be applied down to molecular-sized confinements, although the effective surface tensions used can differ from their macroscopic values.^14^

To investigate the thermodynamic nature of the observed cavitand dewetting transition, we expanded our simulation study to examine the hydration of a wider range of host rim functionalizations over pressures ranging from −500 to 2500 bar.^48^ These particular scenarios can be difficult to recreate experimentally for two reasons. First, it is much easier to “create” a new host *in silico* than it is to actually synthesize it. This is particularly the case with hosts with symmetry lower than *C*_4*v*_, e.g., those with only one unique functional group at the rim of the host (*C*_*s*_). Second, typical methods for guest binding analysis, e.g., NMR spectroscopy, are not routinely set up for high-pressure work. Even if that is the case, modern instrumentation is normally only rated to approximately 14 bar of overpressure.

In addition to hosts **2** and **4**, we studied *in silico* cavitands with one, two, and three methyl groups placed at the *endo*-position. At atmospheric pressure, we found that the probability of observing the dry state (*n* = 0) grows systematically with the number of *endo*-methyl groups, while the wet-state (*n* ≈ 4) probability decreases with increasing *endo*-methyl portal functionalization. In all hosts, we consistently observed a probability minimum at *n* = 1 between the dry and wet states. Wetting/dewetting of all of the cavitand pockets could subsequently be tuned by varying the hydrostatic pressure, with dry states favored with decreasing pressure (or tension in the case of negative pressure) and wet states favored with increasing pressure. Over the entire range of pressures examined, the dry and wet states were separated by a probability minimum at *n* = 1. Based on these observations, we concluded that the hydration of the cavitands corresponded to a two-state-like transition between a dry and wet state. We subsequently developed a capillary evaporation model that quantitatively described the filling of cavitand pockets with water as a function of pressure. Within the context of this model, the role of an *endo*-methyl group is to shift the effective pressure within the host pocket to lower pressures in proportion to their increasing number, thereby stabilizing the dry state. This work highlighted the potential importance of considering pressure effects in water-mediated host–guest association.

## Guest-Mediated Host Dimerization

Cavitand hosts readily scavenge amphiphilic and anionic species to form 1:1 complexes. In the resulting complexes, the portal region of the host–guest complex remains relatively hydrophilic because of the charged headgroup of the amphiphile or charged anionic guest. However, in cases where the guest is devoid of any polar moiety, the portal region of the pocket remains relatively non-polar and the complex can assemble into a capsule. The simplest example of such an assembly is the 2:1 host–guest complexes formed between **2** and steroid guests.^3^ Proton couplings measured via nuclear Overhauser effect ^1^H NMR (NOESY NMR) indicate that the rims of two cavitands face one another, forming a capsular assembly with the guest buried within the inner space of the capsule. If the steroid guest is too long or too wide, for example, cholesterol, the capsular assemblies are unable to fully form. As a result of the two “hemispheres” being unable to clamp down on one another, the complexes have reduced thermodynamic and kinetic stability. A highly preorganized guest is not necessary for capsule formation. For example, simple alkenes form extended or J- and U-shaped “hairpin” motifs within the capsule depending on the length of the guest.^4,49^ Indeed, even propane and *n*-butane can trigger capsule formation. Here, with these small guests, two alkanes are encapsulated to form 2:2 host–guest complexes. As might be anticipated, guest size is key to the thermodynamic and kinetic stability of the complexes; so much so that capsule formation can be used to sequester a stronger binding alkane from the gas phase to affect the separation of hydrocarbon gases.^50^

**Figure 3:**
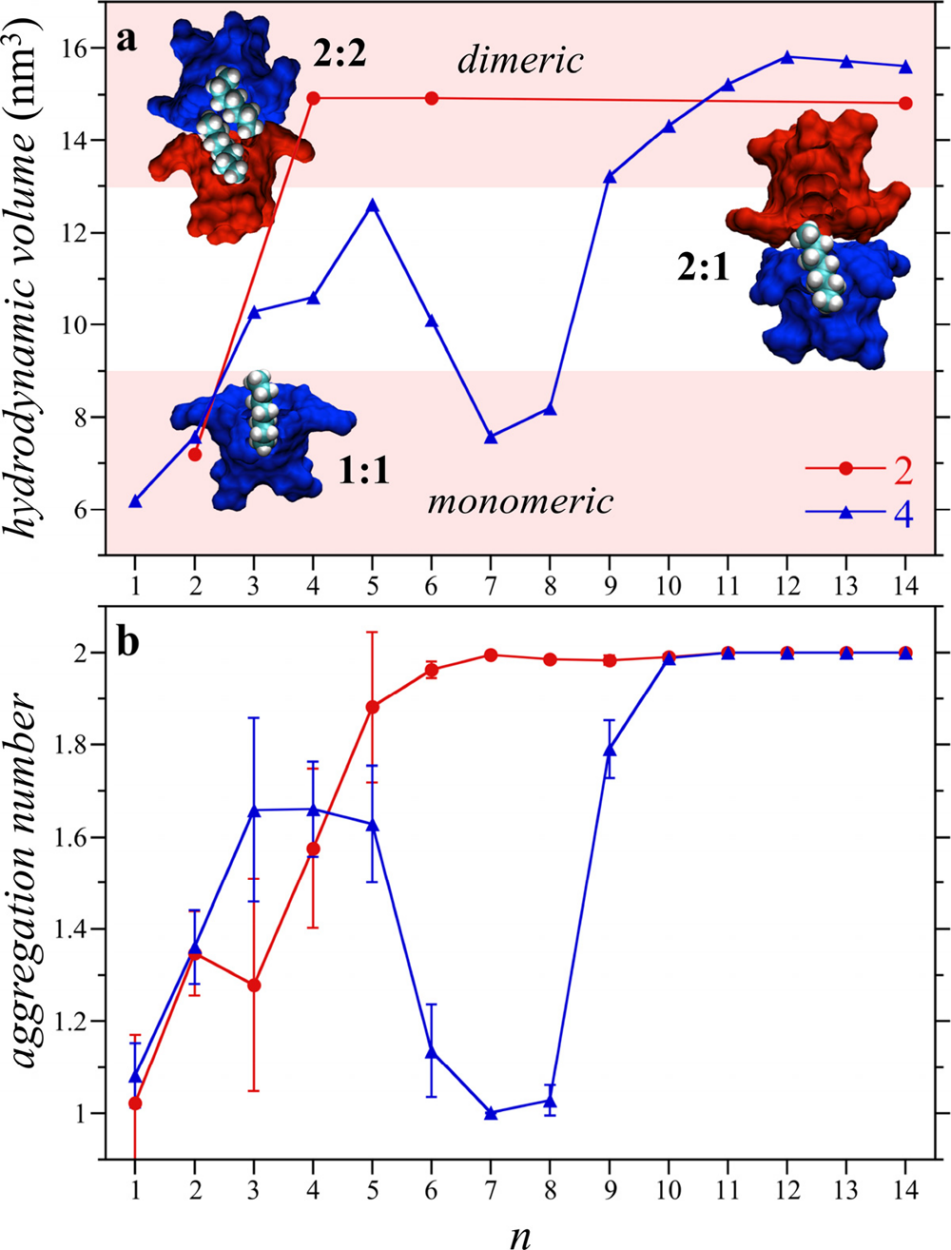
Association of hosts **2** and **4** with alkanes of length *n* to form monomeric 1:1 and dimeric 2:2 or 2:1 complexes in water. (a) Experimental hydrodynamic volumes of host–guest complexes as determined by pulsed-gradient stimulated spin–echo NMR spectroscopy. Cavitand snapshots made with VMD software support.^87^ VMD is developed with NIH support by the Theoretical and Computational Biophysics group at the Beckman Institute, University of Illinois at Urbana–Champaign. (b) Mean host aggregation numbers as determined from simulations and the complex reaction path model. Simulation error bars indicate one standard deviation. Adapted with permission from ref (53). Copyright 2017 American Chemical Society.

A systematic experimental study of the association of hosts **2** and **4** with *n*-alkanes showed distinct assembly patterns with increasing guest chain length (Figure 3a).^51^ Experimentally, the binding of *n*-alkanes is probed by diffusion-ordered NMR spectroscopy (DOSY NMR) which measures the diffusion coefficient (and therefore hydrodynamic volume via the Stokes–Einstein equation) of the assembly by the application of gradient radio-frequency pulse. Methane (C_1_), for instance, readily binds to host **4** to form a 1:1 complex, while it does not evidently associate with **2**. This observation is consistent with host **4** forming stronger complexes with hydrophobes as a result of the comparative ease water is displaced from its pocket.^40^ The next homologue, ethane (C_2_), forms 1:1 complexes with both hosts.^51^ Host **2** forms dimeric complexes with further increases in the guest size beginning with C_3_, as indicated by the drop in the diffusion coefficient of the complex by a factor of 1.25 . Rather than simply forming 2:1 complexes as in the case of the steroids, the alkanes C_3_–C_8_ form 2:2 complexes with **2**. However, between C_8_ and C_9_, the assembly with **2** switches from 2:2 to 2:1 complexes. This 2:1 stoichiometry persists from C_9_ to C_26_, the longest guest examined experimentally.^52^ Similar to host **2**, **4** forms 2:2 complexes for alkanes from C_3_ to C_6_.^51^ The assembly state, however, reverts back to monomeric 1:1 complexes for C_7_ and C_8_ before switching to dimeric 2:1 complexes for guests from C_9_ to C_14_. Even larger tetrameric and hexameric complexes can be formed by host **4** with even longer alkane guests, as discussed further below.

The distribution of hosts and guests between distinct complexes can be rationalized via a reaction network model. The set of reactions describing host assembly with alkane guests (*A*) to form 1:1, 2:1, and 2:2 complexes are
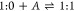
1a
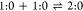
1b
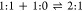
1cand
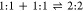
1dNote here 1:0 indicates an empty host. The formation of empty dimeric complexes (2:0, eq 1b) has not been observed experimentally but is considered for the sake of completeness. The equilibrium concentrations of each type of complex (e.g., [1:1]) can subsequently be determined by solving the reaction equilibrium expressions
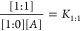
2a
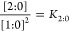
2b
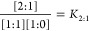
2cand
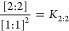
2dExperimental evaluation of the set of equilibrium constants, *K*_*X*__:*Y*_, is hampered by the vanishingly low solubilities of *n*-alkanes in aqueous solution. This precludes host–guest titration experiments used to determine affinities by monitoring the amount of host–guest complex as a function of host–guest ratio. Relatedly, even in the cases where host–guest affinities can be measured from the host–guest ratio, the vanishingly small solubility of the guest again precludes experimental evaluation; only the host–guest complex is typically observed at equilibrium. The alkane guest solubilities and the association free energies between distinct host and guest species following the proposed reaction network, however, can be evaluated from molecular simulations to gain insight into the non-monotonic assembly patterns of host **4** compared to **2**.^53^ Specifically, pairwise association free energies for forming each complex, Δ*G*_*X*__:*Y*_, can be evaluated following the proposed reaction scheme from the potentials of mean force (PMFs) between hosts and guests in water along specified reaction trajectories using molecular simulations. A PMF corresponds to the free energy of a system with two of its components, hosts and guests, at a specified position and orientation relative to them being infinitely far away from one another. The pairwise association free energies were evaluated from the minima in the PMF free energies for bringing hosts, guests, and host–guest complexes together aligned along the rotational axes of symmetry of the cavitands.

**Figure 4:**
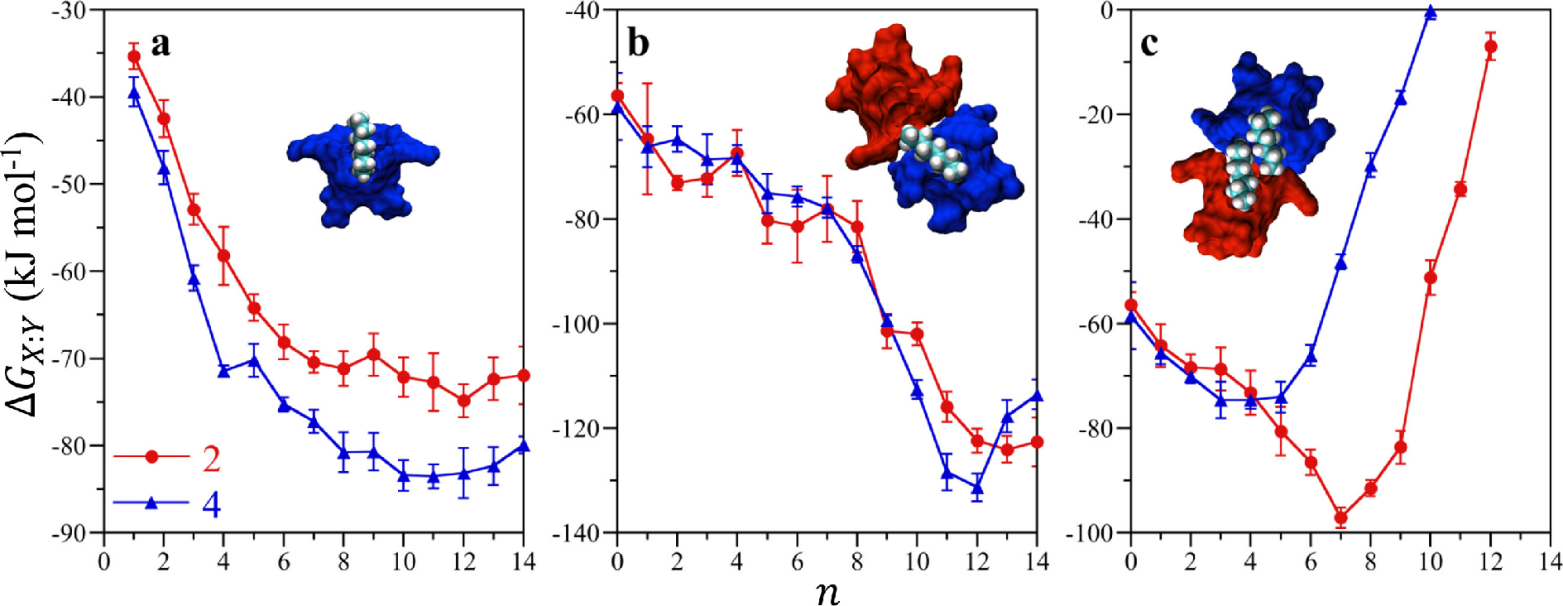
*X*:*Y* complexation free energies between hosts **2** and **4** with alkanes of chain length *n* in water as determined from minima in the potentials of mean force evaluated from molecular simulations. (a) 1:1 host/guest association free energies determined by bringing an empty cavitand (1:0) together with a single alkane. (b) 2:1 host/guest association free energies determined from bringing an empty host together with a 1:1 complex. (c) Host/guest association free energies determined from bringing two 1:1 complexes together. Simulation error bars indicate one standard deviation. Adapted with permission from ref (53). Copyright 2017 American Chemical Society.

Association free energies determined from molecular simulation for forming 1:1, 2:0, 2:1, and 2:2 complexes reported in Figure 4 reveal clear differences between hosts **2** and **4**.^53^ Beginning with methane, the 1:1 association free energies (Figure 4a) for both hosts systematically drop with increasing chain length up to C_6_, after which the free energy effectively levels off. The depth of an individual cavitand pocket is approximately the length of an individual C_6_ chain. Hence, the plateau for longer chains corresponds to the guest size after which the host pocket is filled by one end of the alkane while leaving the remainder exposed to water, thereby gaining no additional benefit from association. The association free energies of alkanes with host **4** are consistently more favorable (more negative) than that of host **2**, attributable to host **4** being drier than host **2**.^40^ The 2:1 association free energies (Figure 4b) for both hosts show that, while dimer formation is favorable for shorter alkanes, the association free energy drops precipitously for guests longer than octane. This drop in the association free energy results from the guest being long enough to span the two hosts. While the 2:1 association free energies for both hosts are nearly the same up to C_11_, host **4** appears to pass through a free energy minimum with increasing guest length for C_12_, while host **2** appears to level out for longer chains. The most significant differences between the two hosts arise for the 2:2 association free energies (Figure 4c). Qualitatively, the 2:2 association free energies of both hosts initially drop with increasing guest chain length before shifting toward positive values beyond a critical chain length. The difference between the two hosts is that the turn toward more unfavorable, positive free energies for 2:2 complex formation occurs at C_7_ for host **2** and C_5_ for host **4**. It may be anticipated then that 2:2 complexes for host **4** are more unstable than those for host **2**.

To understand the origin of the non-monotonic assembly patterns observed for host **4** with increasing guest length, we proposed that the equilibrium constants appearing in eqs 2a are related to the association free energies as
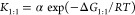
3afor monomeric 1:1 complexes, and
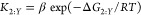
3bfor dimeric 2:*Y* complexes. The prefactors α and β are effective integration constants that result from the multidimensional integral for the equilibrium constants over the full PMF. Since we are limited in our knowledge of the PMF to interactions along a single trajectory, however, we assumed these constants are the same for both hosts and independent of the guests. This assumption can be rationalized by the form of the integral required to evaluate the reaction constants from the full multidimensional PMF along all possible reaction trajectories and orientations.^54−56^ We subsequently fit α and β to the monotonic assembly properties of host **2** to predict the non-monotonic assembly properties of host **4**.

Using the fitted α and β, the simulations accurately reproduce the assembly patterns for both cavitands in excellent agreement with those experimentally determined from NMR spectroscopy (Figure 3b). More importantly, the molecular-level detail available to the simulations permits interpretation of the origin of the non-monotonic assembly behavior of host **4** with alkanes of increasing length. Considering Figure 4, the 2:2 association free energies exhibit the most distinct differences between the two hosts. Specifically, the unfavorable rise in the 2:2 association free energy of host **4** for guests longer than C_5_ destabilizes the complex before the drop in the 2:1 association free energy beginning with C_9_. Resultantly, the 1:1 assembly becomes the dominant complex species observed for C_6_ and C_7_. In the case of host **2**, the rise in the 2:2 association free energy for guests longer than C_7_ nearly coincides with the drop in its 2:1 association free energy, thereby triggering the 2:2 complex transition to the 2:1 complex between C_8_ and C_9_. The non-monotonic assembly of host **4** is therefore a direct result of the destabilization of the 2:2 complex. The premature destabilization of the 2:2 assembly of host **4** results from its four *endo*-methyl units meshing together like gear teeth and constricting the passage between the dimerized cavitands forming the capsule. The 2:2 association free energy of host **4** subsequently rises for alkanes C_6_ and longer chains, due to the guest filling the pocket of a single host while being unable to thread two chains through the narrowed portal between hosts in the dimer. The wider portal of host **2**, on the other hand, permits two alkane guests to simultaneously negotiate the passage between cavitands in a dimer, stabilizing 2:2 complexes for even longer guests than host **4**.

Further simulations were applied to investigate the role of partial *endo*-methylation of host portal rims in regulating cavitand assembly.^57^ Specifically, the assembly of mono-, di-, and tri-*endo*-methylated cavitands with alkanes was simulated to systematically bridge between hosts **2** and **4** by considering partial constriction of the passage between hosts in a dimeric capsule. The methyl groups were found to moderate the assembly equilibrium between dimeric and monomeric complexes, with monomeric 1:1 complexes with C_7_ and C_8_ systematically growing in stability as the extent of host methylation increases. While in principle these partially methylated hosts can be synthesized, the reaction products will be a statistical distribution of portal methylations that cannot be easily purified.

Experimentally, the stoichiometries of the assemblies formed between hosts **2** and **4** with alkanes diverge as the guest chain length increases. While host **2** only forms 2:1 complex species for alkanes up to C_26_, host **4** can form tetrameric 4:2 and hexameric 6:3 complexes (*vide infra*).^58^ These distinct assembly pathways can ultimately be attributed to the steric interactions between the *endo*-methyl groups of the cavitand subunits which decrease with the larger assemblies as the bite angle between any two adjacent cavitands increases.

## Guest Packing within Octa-Acid Dimers

To experimentally probe the conformational motifs of guests packed within dimeric capsules of host **2**, two-dimensional ^1^H–^1^H correlation NMR spectroscopy (COSY NMR) was performed in the solution state. It was previously determined by Rebek and co-workers that the change in chemical shift (Δδ, ppm) of the guest proton signals between the free and bound states can indicate how deep a proton of a guest lies in a conical cavity such as velcrands and deep-cavity cavitands.^59,60^ The difference and signal anisotropy in the bound state is brought about by the structure of the cavitand itself; walls composed of π systems physically and magnetically shield the guest from the external magnetic field causing a spread of signals.^61−63^ Sections of the guest that are deeper in the cavity have larger negative Δδ shifts than those guest protons that are closer to the equator of the capsule, and thus the portal of the cavitand. These experiments reveal that for C_9_–C_16_ the guest assumes a conformation such that the terminal methyl protons are deep in the pocket of the opposing cavitands, with the intervening methylenes progressively compressed within the equatorial region of the capsule and the guest in part adopting a motif that ranges from fully extended in C_9_–C_11_ where all *gauche* interactions are minimized to a form akin to a compressed α-helix in C_12_–C_16_. In contrast, because of the limited compressibility of the guests within the capsule, guests C_17_–C_23_ are forced to adopt a hairpin (or J/U-shaped) motif in the capsule: one end of the molecule resides in the depths of one pocket, while the other end lies in the vicinity of the equatorial region, and the end of the other pocket contains the turn. From C_24_ to C_26_, the guest motif again switches—both methyl groups again reside deep in the interior poles of the capsule, while the intervening methylenes coil to adopt a disk-shaped conformation to give the guest an overall spinning-top-like form. In such cases, the central methylene groups of the guest can “hemorrhage” out of the equator of the capsule and be exposed to free solution. These different ways that guests can pack within the dimeric capsule can be taken advantage of to control the chemical properties of bound guests. For example, it has been shown that the bound guest motif can greatly influence the acidity and rate of cyclization of internalized guests (see below).^64^

A complementary molecular simulation investigation^65^ was conducted to determine if the predicted succession of bound guest conformational motifs conformed to those inferred by NMR spectroscopy and to interpret the role of guest packing within the interiors of host capsules on determining the resultant guest conformations observed. Given the restricted environment within which guests are confined, simple molecular simulations at a fixed temperature may not be able to sample the ensemble of potentially available conformers. To attempt to overcome this difficulty, alkanes from C_9_ to C_25_ were initially simulated within a host **2** capsule in a vacuum using replica exchange molecular dynamics to sample conformations over a broad temperature range to hopefully overcome free energy barriers between the initial and equilibrium guest conformations. In these solventless simulations, the capsules were held closed using harmonic restraints. Following this equilibration phase, the host–guest complexes were placed in aqueous solution, the harmonic restraints removed, and long simulations performed to allow the conformationally equilibrated guests to relax and explore conformations in the unrestrained, hydrated conformations.

**Figure 5:**
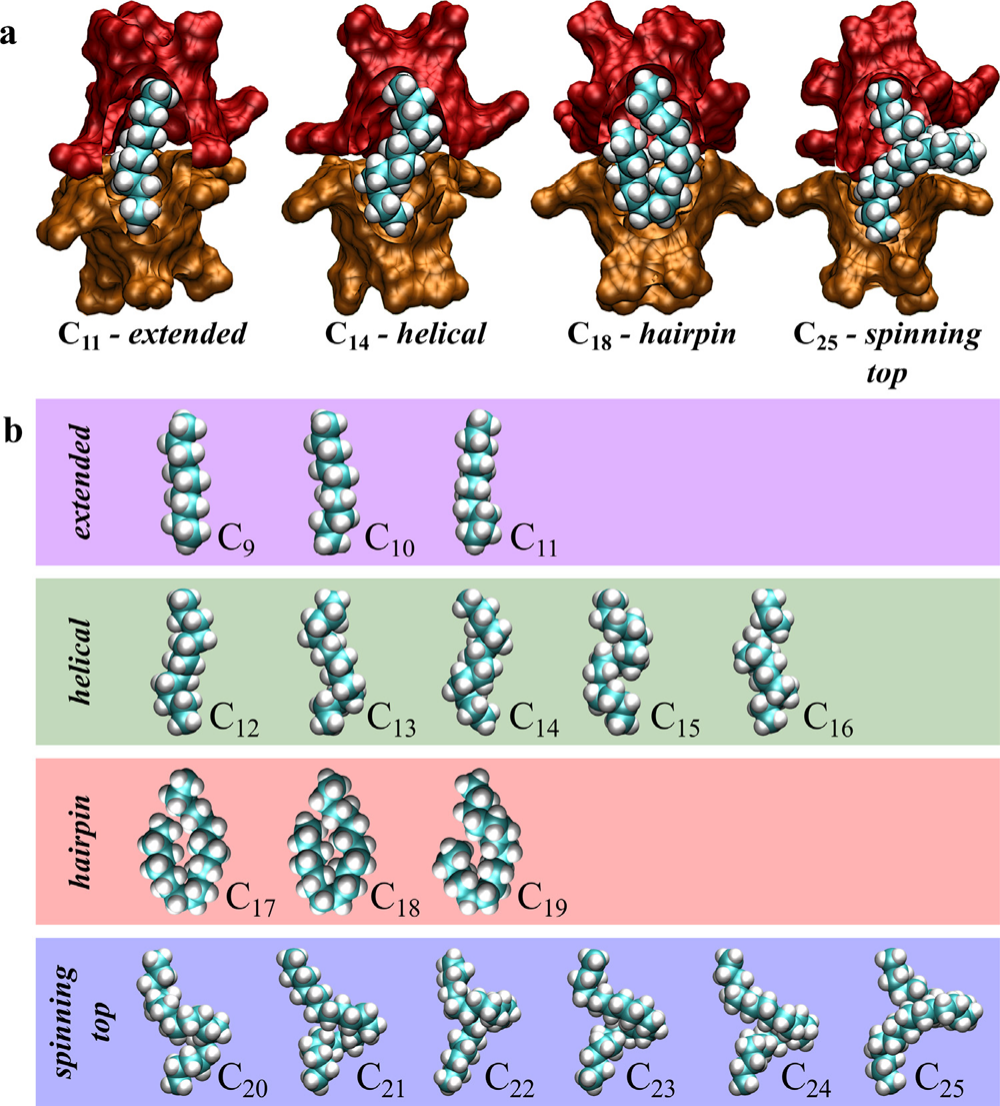
Succession of dominant guest conformational motifs encapsulated within a host 1 dimer for alkanes from C_9_ to C_25_. (a) Representative snapshot of the extended (C_11_), helical (C_14_), hairpin (C_18_), and spinning-top (C_25_) motifs within a host **2** capsule. The two cavitands are represented by the red and orange surfaces, while the alkanes are illustrated using the van der Waals representation. The front of the capsule has been cut away from this image to more clearly view the guests. (b) van der Waals representation of the succession of conformational motifs observed with increasing guest length. The host capsule has been omitted for clarity. Cavitand snapshots made with VMD software support.^87^ VMD is developed with NIH support by the Theoretical and Computational Biophysics group at the Beckman Institute, University of Illinois at Urbana–Champaign. Adapted with permission from ref (65). Copyright 2016 American Chemical Society.

As inferred from NMR spectroscopy, our simulations found that the dominant guest conformations progressed from an extended, to helical, to hairpin, to spinning-top motif with increasing chain length (Figure 5a). These snapshots illustrate the crowding of the guests within the host capsule. Indeed, the integrity of the capsule is disrupted for C_25_ so that the turn in the spinning-top motif pries the two cavitands apart and partially herniates from the capsule. This observation highlights the importance of relieving the harmonic restraints from our vacuum equilibration simulations to properly capture the guest conformation. These conformational motifs systematically follow one another with increasing alkane length, as can be seen in the succession of the dominant conformations observed from C_9_ to C_25_. While the agreement with experiment is excellent, we find that the simulations do predict the transition from the hairpin to spinning-top motifs for shorter chains (between C_19_ and C_20_) than experiment (between C_23_ and C_24_). Nevertheless, shifts in the ^1^H-NMR chemical shifts of the alkane protons upon transfer from solution to the host environment predicted by gauge invariant atomic orbital^66^ calculations performed using Gaussian^67^ on the dominant simulation conformations reported in Figure 5b are strongly correlated with those determined experimentally.^65^ These simulations subsequently support the experimentally inferred progression of guest conformers encapsulated within host **2** dimers.

Beyond reproducing the experimental sequence of alkane conformational motifs, we were able to calculate the internal strains on the guests that dictate the conformations observed. Specifically, we evaluated the incremental free energies of extending the encapsulated alkanes by one methylene unit from C_9_ to C_25_, Δ*G*_*n*__→*n*+1_. For the shortest chains considered, from C_9_ to C_12_, the free energy increment was found to be negative and constant, with Δ*G*_*n*__→*n*+1_ ≈ −7 kJ mol^–1^. This is consistent with those guests both favoring transfer into the capsular environment and adopting the same extended conformational motif. Between C_12_ and C_16_, the extensional free energy increment consistently increased by +2 kJ mol^–1^ with increasing chain length, such that for C_16_ and C_17_ the free energy ultimately becomes positive with Δ*G*_16→17_ = 2.5 kJ mol^–1^, comparable to the thermal energy *RT*. The increase in the free energy over this chain length regime results from the guests adopting an increasing fraction of *gauche* dihedral conformations in the helical motif that are energetically more unfavorable than the *trans* conformation that dominates the extended motif. As suggested by the 1:1 complexation free energies reported in Figure 4a, the depth of the host **2** pocket is comparable to a C_6_ chain. It therefore makes sense that the transition to the helical motif initiates with C_12_ at which point the guest spans the length of the dimeric capsule. The increase in the extensional free energy is then analogous to the compression of a spring as the host capsule attempts to accommodate guests of increasing length. Once the extensional free energy becomes positive, the guest adopts the hairpin motif to minimize the *gauche* strain along the entire length of the chain and localize the unfavorable *gauche* conformations at the hairpin turn. Resultantly, the extensional free energy between C_17_ and C_20_ is approximately constant and positive, equal to the Δ*G*_16→17_ increment. Eventually, beginning with C_20_, the guest fills the inner space of the capsule and wedges the two cavitands apart, herniating the guest (Figure 5a) and partially exposing it to water. At this point, the guest adopts the spinning-top motif, which corresponds to a hairpin that has splayed its two ends into the opposing hosts in the dimer. Beginning with C_21_, the extensional free energy drops to −4 kJ mol^–1^ for further increases in the alkane length. While negative, this free energy increment is less than that observed for growing the extended motif, reflecting the partial exposure of the guest to water in the spinning-top motif. In this motif, the splayed guest hairpin extends its two terminal hexyl groups into each host, while the remainder of the chain is extruded between the rims of the hosts, forming a looping turn. In addition to demonstrating the strain in the encapsulated guests, these calculations highlight the role of the depths of the two cavitands in the dimer on selecting the preferred guest conformation. It also suggests that, if the walls of the cavitand can be made deeper, the conformational transitions will shift to increasing chain lengths.

## Host Assembly into Multimeric Complexes

The narrower portal of host **4** restricts guest conformations within dimeric 2:1 complexes. Most notably, the inability of two alkane chains to thread the passage between cavitands of a host **4** dimer as discussed above bars the formation of a hairpin motif. Experiments using DOSY NMR corroborate this claim. While the binding of hydrocarbons C_1_–C_14_ to **2** shows a monotonic trend from a monomeric host–guest complex (C_2_) to dimeric, capsular complexes (C_3_–C_14_), this trend does not completely apply to **4**. The DOSY spectra of guests C_1_, C_2_, C_7_, and C_8_ all indicate the formation of monomeric 1:1 complexes. Guests C_9_–C_14_, on the other hand, were determined to complex to dimers of **4**, whereby the hydrophobic surface of the guest is completely encapsulated. An expansion of the complex is seen as the guest size increases to C_12_–C_14_: the guest becomes large enough that the void space is not only completely filled, but also the two halves of the capsule are pushed farther apart in order to accommodate the increase in guest volume. Guest C_5_ presented an anomaly such that it formed a 2:2 complex. This phenomenon can be explained by the fact that dimerization of **4** leads to a less stable capsule than that formed by **2**. As a result, the capsule is more particular about how guests bind. Thus, small (C_1_ and C_2_) and medium sized guests (C_7_ and C_8_) form 1:1 complexes because their respective 2:2 and 2:1 host–guest complexes would contain too much empty inner space. Contrastingly, the C_5_ guest, and C_9_ and bigger guests, can nicely fill the capsule containing two guests or one guest, respectively. Intermediary guest lengths, i.e., C_3_, C_4_, and C_6_, however, are caught between these two possibilities. As a result, each guest exists as a dynamic equilibrium of 1:1 and 2:2 host–guest complexes.

With bigger *n*-alkanes, TEMOA **4** was found to form 4:2 and 6:3 host–guest complexes.^58^ Thus, using ^1^H and diffusion NMR experiments, it was shown that, whereas C_14_ formed the dimeric complex, C_17_ formed the tetrameric 4:2 species and C_24_ the hexameric 6:3 host–guest complex. An analysis of the inner space of these larger complexes noted that both were more capacious than simply double or triple the volume of the dimeric capsule. The inner volume of the tetrameric capsule is equal to four cavitand volumes, plus a central pseudotetrahedral volume enclosed by the cavitands, whereas the hexameric capsule has a total volume of six cavitand volumes plus a core, pseudocubic defined by the hexahedral arrangement of the cavitands (see below). This work did not definitely identify the driving forces for the tetrameric and hexameric assemblies, but as OA **2** does not form tetrameric and hexameric assemblies, it is evident that the four *endo*-methyls of TEMOA **4** are key. As noted above, the bite angles between adjacent cavitands in the dimer, tetramer, and hexamer are approximately 0, 70, and 90°, respectively, and models suggest that angles greater than approximately 30° reduce the steric clashes between rim methyl groups in opposing cavitands that are prevalent in the dimer interface. As a result, whereas OA **2** has a predisposition to form a dimeric capsule, TEMOA **4** is more predisposed to form higher assemblies when guests are suitably large enough to fill the inner space.

**Figure 6:**
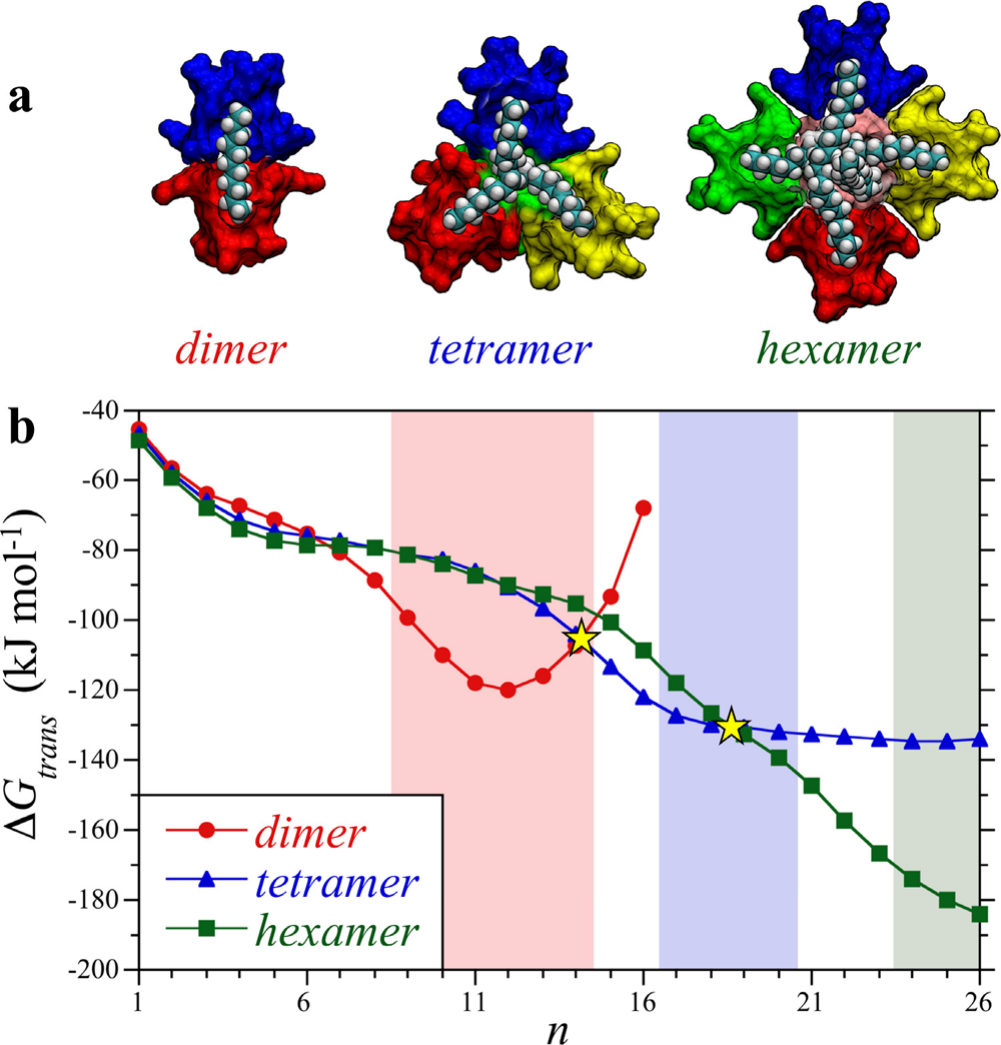
(a) Simulation snapshots of guests packed within host **4** dimeric, tetrameric, and hexamer complexes. Part of the host complex structure is removed to show the guests within the complex interior. The guests are illustrated by the van der Waals surfaces, while the individual cavitands are represented by different colored surfaces. The host portals in the tetramer are placed on the faces of a tetrahedron, while those in the hexamer are placed on the faces of a cube. Cavitand snapshots made with VMD software support.^87^ VMD is developed with NIH support by the Theoretical and Computational Biophysics group at the Beckman Institute, University of Illinois at Urbana–Champaign. (b) Free energies of transferring alkane guests of length *n* from the vacuum to the interior of a preformed host dimer, tetramer, and hexamer complex. While one, two, and three guests are transferred into the dimer, tetramer, and hexamer, respectively, the free energies are reported on the basis of an individual chain to facilitate comparison. The simulation symbols are defined in the figure legend. The yellow stars indicate crossing points between the dimer, tetramer, and hexamer transfer free energies. The red, blue, and green shaded background regions indicate the experimental ranges of stability of the dimeric, tetrameric, and hexameric complexes, respectively. Reproduced with permission from ref (68). Copyright 2018 The Royal Society of Chemistry.

While our analysis of the non-monotonic assembly of host **4** was amenable to direct simulations of the interactions between guest and host subunits, the number of species coming together to form tetramers (4 hosts and 2 guests) and hexamers (6 hosts and 3 guests) complicates reduction of multimer formation into simple pairwise processes (Figure 6a). A conclusion that can be drawn from our simulations of dimer assembly and the encapsulated guest conformations is that guest packing the confined spaces of the complex plays a significant role in determining the structures formed. Rather than consider the full assembly process then, we performed simulations to evaluate the free energies of transferring alkane guests from a vacuum into the interiors of preformed dimers, tetramers, and hexamers of host **4** in solution.^68^ Given that these complexes are stable only over a limited range of guest sizes, the host structures were held fixed by harmonic constraints to ensure free energies could be evaluated over the full range of guest sizes. Alkanes from C_1_ to C_26_ were considered. The number of guests simultaneously transferred into a complex was such that the host-to-guest ratio was 2:1, in line with the experimental complex stoichiometry.

The guest transfer free energies determined from simulation reported in Figure 6b show distinct preferences for different complex morphologies with changing alkane chain length. For alkanes from methane to hexane, the guest transfer free energies into each complex are all approximately the same, showing no clear preference. As established above (Figures 3 and 4), however, alkanes shorter than nonane prefer either 1:1 or 2:2 complexes over the 2:1 complex. Given the greater entropic penalty that may be anticipated for forming tetrameric and hexameric complexes, we believe neither of these complexes will be stable. Examining the simulation trajectories, we find that the shorter guests are largely segregated into individual host pockets away from one another. The similarities of the transfer free energies in this regime are then a result of the similarity of the environments each guest explores. For guests longer than hexane that can extend beyond a single pocket, preferences begin to emerge. Specifically, the transfer free energy into the dimer falls below that of the tetramer and hexamer starting with C_7_, dropping to a minimum at C_12_ before rising with further chain length increases. We may then presume that guest packing prefers the dimer over this range of guest sizes, although when we consider competitive equilibrium with the 1:1 and 2:2 complexes, the 2:1 complex does not become dominant until C_9_. Comparing the guest transfer free energies into the dimer against the host **4** 2:1 formation free energies in Figure 4b, we find that the shapes of these curves are practically the same, differing by a free energy shift associated with the contributions from the association of the empty host cavitands. This comparison supports the assumption that guest packing guides assembly. With further increases in the guest chain length, the transfer free energy into the tetramer eventually falls below that into the hexamer (beginning with C_12_) and intersects that into the dimer between C_14_ and C_15_, after which transfer into the tetramer is favored. Eventually, the transfer free energy into the tetramer reaches a plateau, after which the transfer free energy into the hexamer crosses that of the tetramer between C_18_ and C_19_ to become the favored complex morphology.

While the guest–transfer free energies show clear preferences for complexes with increasing numbers of hosts with increasing chain length, the chain lengths for which the transfer free energies between successive complexes size cross one another are shorter than experimentally observed (indicated by the shaded regions in Figure 6b). We attribute this difference to two sources. First, as noted above, the complexes were held rigidly fixed by harmonic constraints during our simulations to stabilize their structure. This constraint diminishes the possibility for the complexes to relax under guest packing strain, increasing the free energy of longer guests and shifting free energy cross points to shorter chain lengths. Second, only considering the guest transfer free energy neglects significant contributions to the assembly process, like the free energy of assembling an empty host complex. Nevertheless, given the reasonable agreement between experiment and the guest transfer free energies, it is reasonable to conclude that guest packing within complex interiors plays a dominating role in determining the stability of the complexes observed.

We may subsequently ask, why do guests of increasing length prefer larger complexes of host **4**? Rather than growing in proportion to the number of hosts, the internalized volume available to the guests in a complex grows faster. For example, while the average interior volume of the dimer is 587 Å^3^, largely associated with the volume of two host pockets, the interior volume of the tetramer is 1920 Å^3^, some 746 Å^3^ greater than that of two dimers. This increment arises from the excess volume bounded by the four hosts at the center of the tetramer complex. The excess volume in the case of the hexamer is 2260 Å^3^. Evaluating the packing of guests within complex interiors, empirically we found that the dimeric, tetrameric, and hexameric complexes are stabilized when the guest packing fraction is 30% or greater. Thus, we conclude the transitions occur when the guest packing fraction is sufficient to stabilize the host structure. While octamers have not been observed experimentally to date, our empirical criteria suggest guests C_35_ and longer would be required to stabilize this complex. Given that C_35_ does not melt until 75 °C, octamers would need to be prepared at even higher temperatures and perhaps higher pressure to minimize solvent evaporation.

## Host–Guest Binding Models for Benchmarking Drug Binding Affinity Predictions

A large fraction of the time and cost of drug development involves hit screening, lead optimization, and primary assays. This monetary and temporal cost would be significantly lowered if it were possible to predict high-affinity ligands for targets. The statistical assessment of the modeling of proteins and ligands (SAMPL) challenges are a set of collaborative analysis–computation experiments designed to improve the predictive power of computational drug design.^69,70^ These challenges serve as benchmarks for the determination of protein–ligand affinities via the modeling of analogous small-molecule systems. A question regarding binding affinity predictions, however, is whether errors arise from the accuracy of the force fields used and/or simulations sufficiently account for the flexibility of the protein backbone. Given the rigidity of cavitand hosts compared to proteins, they provide a route for minimizing one of these sources of potential error to help advance the development of accurate binding affinity prediction protocols. In the most recent iteration of the challenge (SAMPL7), we showed that moving the carboxylate groups at the rim of **2** closer to the portal of the weakly solvated cavity, i.e., *exo*-octa acid **5**, changes the binding properties of the host.^71^ In the case of **2**, unexpectedly, negatively charged guests bound slightly more strongly than positively charged ones. This was reversed in the case of *exo*-octa acid **5**. Here, positively charged guests bound more strongly than negatively charged ones. However, this difference was only 70% of what might be expected from Coulombic (ion–ion) considerations. Moreover, for the four negatively and four positively charged guests examined, binding to *exo*-OA **5** was always weaker than that to OA **2**. We also used molecular dynamics simulations to show that, because of the proximity of the negative charges in *exo*-OA **5**, its pocket had a higher desolvation penalty.^41^ Thus, both direct ion–ion interactions between host and guest and indirect ion–dipole interactions (between host and water) affect guest affinity. Further molecular dynamics simulations also pointed to changes in water hydrogen bonding as a complex is formed; hence, we believe that differences in guest affinity between the two hosts may also involve the asymmetry of how groups of opposite charge are solvated. Further work on these types of systems is required to parse out all of the factors controlling guest affinity.

## Anion Binding to Cavitand Hosts

**Figure 7:**
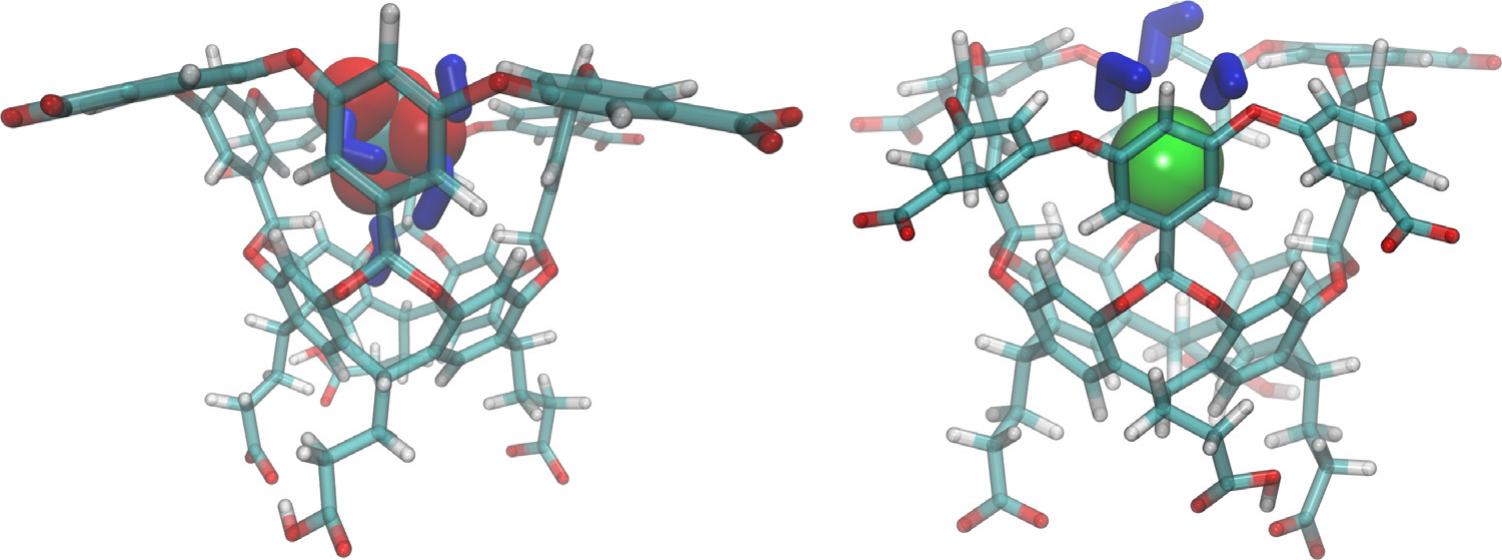
Simulation snapshot of ClO_4_^–^ (left) and I^–^ (right) bound to host **2** illustrating the partial retention of each ion’s hydration shell upon binding. The host is indicated by the licorice structure colored following the convention: hydrogen - white; oxygen - red; carbon - cyan. The anions are illustrated using their van der Waals surface, and hydration shell waters are indicated by the thick dark blue licorice structures.

In a series of papers, the Gibb group demonstrated that the weakly hydrated pocket of **2** can bind charge-diffuse anions. Modeling with the Rick group demonstrated that anions such as I^–^, PF_6_^–^, and ClO_4_^–^ were bound in partially hydrated states (Figure 7).^72−74^ Salts of these anions are at the “salting-in” side of the Hofmeister series and are weakly hydrated. It is believed that this weak solvation is key to them being able to bind to non-polar cavities. More recently, the same group has shown how weakly solvated anions bind to preorganized protuberances on the surface of the protein ubiquitin, particularly at β-turns, suggesting a significant role for anion binding in inducing the salting-in of proteins above their pI value and salting-out below their pI value.^75^

Evidently, the key to anion binding is a weak and flexible solvation shell. Thus, measurements of complexation thermodynamics using ITC showed that the binding free energies of these salting-in anions are enthalpically favored but entropically penalized (Δ*H* < 0, Δ*S* < 0).^74^ Thus, despite that both the host and the guest are formally anionic, complexation is exothermic. On the other hand, smaller anions such as Cl^–^ that are more strongly solvated do not bind. Host–anion interactions in the complex cannot compete with the strong water–anion interactions of the free ion.

Compared to OA **2**, most ion binding to TEMOA **4** is comparable or stronger.^76^ Indeed, in some cases, anion binding is stronger by 1 order of magnitude. Notable examples include PF_6_^–^ (*K*_a_^4^/*K*_a_^2^ = 6.6) and ReO_4_^–^ (*K*_a_^4^/*K*_a_^2^ = 7.2). The most likely explanation for this is that the dryer pocket of **4** means that anions do not have to compete with water to bind. Indeed, calculations of the electrostatic fields exerted by the charges of both hosts found them to be indistinguishable, pointing toward solvation differences between the two hosts playing a controlling role.^76^ However, exceptions to cavitand **4** being a better host seem to present some questions. For example, the hydration free energies of ClO_4_^–^ and ReO_4_^–^ are very similar,^77^ yet in contrast to the date for the latter, the former bound more weakly to host **4** than to host **2**. Clearly other factors must be in play. We suspect that another key factor is the size and symmetry of the ideal solvation shell around an anion. If two anions are similar in size and have similar overall solvation free energies, then the difference in host affinity may be caused by the ability (or inability) of the host to also accommodate the three or four waters of solvation that necessarily must accompany the bound anion.

## Host Capsules and Yocto-Liter Reaction Vessels

Synthetic cavities with weakly solvated pockets have been of particular research interest of late, particularly those that exhibit protective properties for bound guests or function as catalysts that have the potential to mimic enzymes. Among a whole host of possibilities, reactive reagents like white phosphorus can be encapsulated in a supramolecular cage,^78,79^ water-sensitive gold-catalyzed reactions can be performed,^80^ and proton-mediated terpene cyclizations can be achieved—all in water^81−84^ and within the hydrophobic interior of synthetic supramolecular assemblies. Contributing to this growing field, the binding of cyclable α,ω-difunctionalized alkyl chains into dimeric capsules of **2** has been explored.^85^ The alkyl chain guests were composed of α,ω-thioalkyl bromides, which in free solution form polymer rather than cyclize to form the corresponding thioether. However, trapped within the inner space of the capsules, an individual guest cannot take part in polymerization. Rather, the forced proximity of the nucleophilic thiol and electrophilic methylene halide (−CH_2_X) promotes intramolecular cyclization.

To probe the effect of guest packing and host electrostatic potential field, the Gibb group investigated the cyclization of α,ω-thioalkyl bromides of different lengths within a capsule formed by OA **2** and a positively charged analogue assembled from the dimerization of so-called positand **6** (Figure 1). With respect to the guest length, the investigation revealed that guests too long for the capsule that were forced to adopt a J-motif possessing a reverse turn in the main chain (e.g., C_14_) underwent fast cyclization. In contrast, shorter guests (e.g., C_12_) that bound in an extended motif in which each end of the capsule was occupied by one of the termini of the guest underwent a very slow reaction. Reaction rates that differed by 3 orders of magnitude were seen for these two lengths of guests. Interestingly, intermediate reaction rates were observed for very long guests. These too adopted J-motifs within the capsule, but the limited remaining free space within the capsule attenuated cyclization rates considerably. Thus, the capsules demonstrated excellent selectivity for cyclizing guests of lengths C_14_–C_16_.

When rates of cyclization in the positive and negatively charged capsules were compared, it was evident that the transition state to cyclization possessed considerable negative charge. Thus, the half-life of a C_12_ guest was over 3 orders of magnitude shorter in the capsule formed by **6**. An Eyring analysis revealed that both intermediate sized guests and reaction in the capsule formed by **6** were favored by lower enthalpies of cyclization.

A follow-up report examining the p*K*_a_ of simply long-chain thiol guests within the same two capsules revealed three factors that controlled the acidity of a thiol guest. In order of decreasing importance, these were the following: guest motif, host charge, and the nature of the host counterion or exogenous salts in solution. Briefly, when the guest adopted a J-motif in which the thiol headgroup was located at the equatorial region of the capsule, the p*K*_a_ was lowered by up to five units relative to guests that adopted extended conformations where the thiol group was deeply buried. As there were only small differences in the electrostatic potential field at these two points of the inner space (see below), partial solvation of the equatorial located thiol was presumed to be key to making these thiol complexes more acidic. With regard to the nature of the capsule, p*K*_a_ values were generally between one and three units lower within the host **6** capsule. It is understood that these two factors—motif of guest and nature of capsule—contributed to the spontaneous cyclization of the C_14_ α,ω-thioalkyl bromide (J-motif) within the positive capsule; a low p*K*_a_ meant the guest spontaneously deprotonated (and cyclized), whereas, with the other host–guest combinations, the addition of excess base was required to initiate reaction. Finally, it was found that the nature of exogenous salts also affected the p*K*_a_ of bound guests. Salts in which one of the ions could exchange with the counterion of the capsule and condense on its surface raised the p*K*_a_ of the bound guest by attenuating the electrostatic potential field of the host.

**Figure 8:**
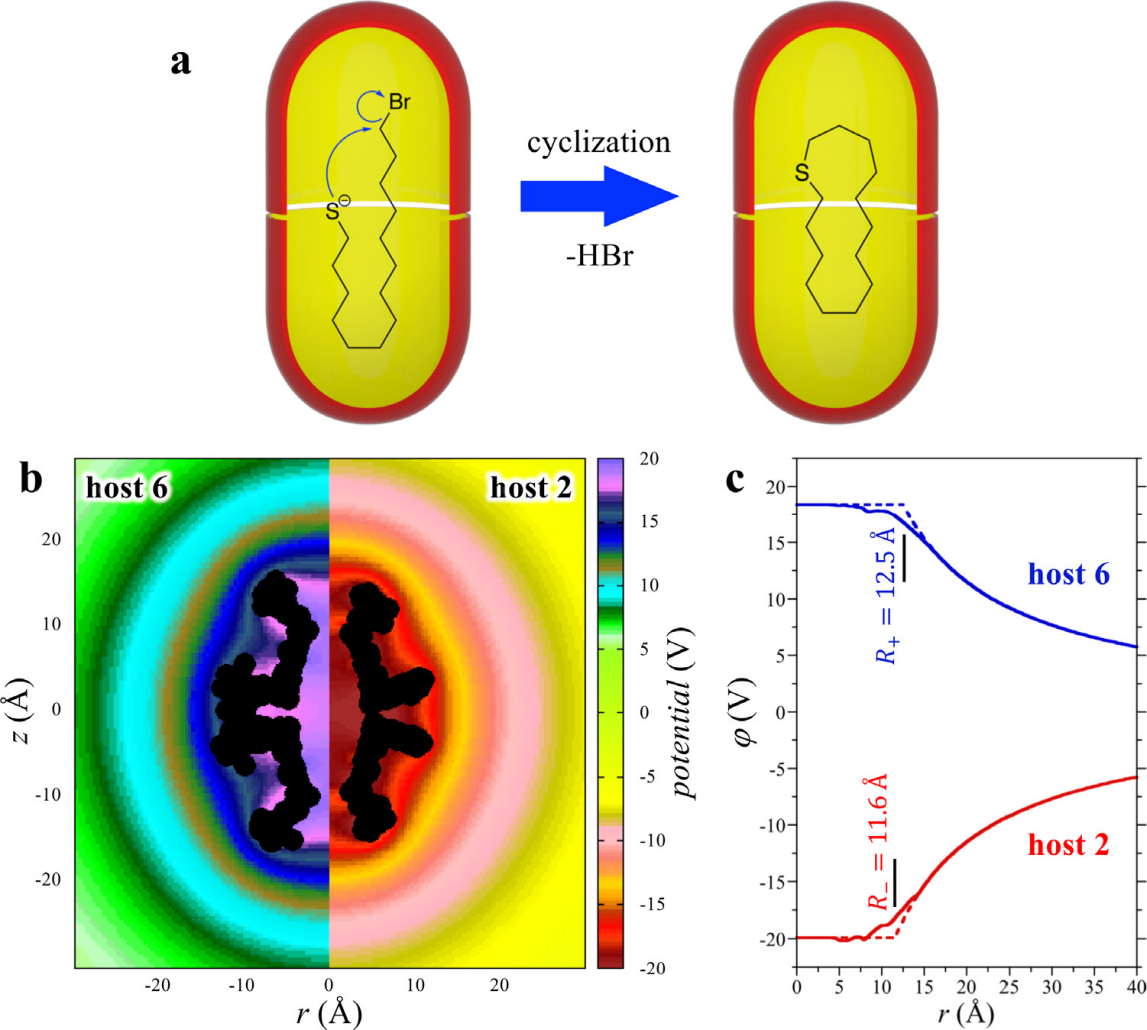
(a) Illustration of the bromo-thiol cyclization reaction within a cavitand capsule with the reaction exhibiting a negatively charged transition state (left). (b) Calculated electrostatic potential distribution about a positand (left) and OA (right) host capsule in a vacuum cylindrically averaged about the C4-axes of symmetry of the two cavitands. (c) Electrostatic potential distribution about the positiand and OA host capsule in a vacuum spherically averaged about each capsule’s center of mass. The full lines indicate the isotropically averaged field about the host capsules reported in part b, while the dashed lines indicate the electrostatic field about a spherical volume with the capsule charge uniformly smeared across its surface. Adapted with permission from ref (64). Copyright 2019 American Chemical Society.

Returning to the cyclization of α,ω-thioalkyl bromide guests within the capsule, complementary potential calculations were performed to probe the role of electrostatic interactions within capsules of hosts **2** and **6** on promoting the cyclization of the guests (Figure 8b). Unsurprisingly, the anionic host **2** generates a negative electrostatic potential field about and within the cavitand, while the cationic host **6** generates a positive field. Perhaps more interesting, the fields within both host capsules were found to be nearly constant, nearly independent of position with the capsule interior. This positional independence is more readily seen if we consider the electrostatic field as a function of distance isotropically averaged about the center of mass of each capsule (Figure 8c). In this case, the electrostatic potential is constant from the center of the capsule to a radial distance of ∼8 Å. Given that this distance lies outside the interior volume of the cavitand, we can conclude the encapsulated guest effectively experiences a constant electrostatic field. Following a transition region from ∼8 to 12 Å, the isotropically averaged electrostatic potential field outside the cavitand dies away as 1/*r*, as expected by Coulomb’s law. The isotropically averaged field about the host capsules can be modeled to a first approximation as that generated by a spherical volume with a uniform charge (equal to the charge of the capsular complex) smeared across its surface area (Figure 8c). This model (with a radius of 12 Å) accurately captures both the internal and external fields of the capsules, deviating only in the transition region between these regimes. Assuming the host capsule can be modeled as a sphere, the effect of placing a charge at the center of each host, mimicking deprotonation (charging) of the guest, can be evaluated within the context of the linearized Poisson–Boltzmann equation to account for the effects of low concentrations of added salt. The free energy difference between placing a charge within the cationic and anionic capsules is subsequently determined as
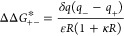
4where *δq* (= –1 e) is the charge on the guest within the capsule, *q*_–_ (= –16 e) and *q*_+_ (= +16 e) are the net charges on the anionic and cationic capsules, respectively, ε (= 80 for water) is the dielectric constant of the solvent, *R* (= 12 Å) is the effective Born diameter of the host capsules (assumed here to be given by the mean isotropic radii in Figure 8c), and κ is the inverse Debye length determined by the added electrolyte concentration in solution (200 mM NaOH in these calculations). The free energy difference calculated from this expression is ΔΔ*G*_+–_^*^ = 17.2 kJ mol^–1^, which is in reasonable quantitative agreement with the p*K*_a_ shifts of the alkylthiol between the anionic and cationic capsule environments and the ∼10^3^-fold increase in the thioalkyl bromides cyclization reaction rate increase that passes through an anionic intermediate. While the agreement between experiment and theory is surprising given the approximations made to arrive at eq 4, we believe this back of the envelope calculation demonstrates that electrostatic stabilization of the anionic guest within the cationic complex over the anionic complex plays a dominating role in determining the impact of the host on the experimental observations.

## Outlook

The essence of a successful collaboration is the ability of researchers to come together to address problems from different points of view to arrive at new insights that could not have been arrived at alone. As recently discussed in a review by Cremer et al.,^88^ the forays of supramolecular chemists from organic media into aqueous solutions represent a rich field of opportunity for synthetic and physical chemists to collaboratively explore, bringing to bear a deep understanding of experimental host–guest interactions and theoretical insights into the role of the solvent on driving association. While our initial collaboration focused on using simulations to try to understand prior experimental observations, such as understanding the conformational motifs of guests within capsules and the non-monotonic assembly patterns of host–guest complexes, more recent work has seen simulation predictions drive the experimental efforts, as was the case for our study of the wetting/drying of cavitand pockets and its impact on host–guest association. As the collaboration has matured, both experimental and theoretical questions have driven richer questions regarding host–guest complexation phenomena in aqueous solutions. Ongoing research questions we are exploring include the following: What is the impact of polarizability on driving anion binding to cavitand hosts? What is the extent to which the described electrostatic potential field of capsules can affect reactivity in general? How might co-nonsolvency and macromolecular crowding impact complex formation for weakly interacting cavitands? What role might pressure play on moderating association? Answering these and other such questions has the potential to expand our molecular-level understanding of different non-covalent interactions in water, point to new technologies such as sensors or purifiers that rely on the orchestration of these forces, as well as shine light on the function of biological systems. As water is the solvent of life, and therefore the greenest of media, the opportunities are boundless.
